# Supplementary material for: Characterization of a Toxoplasma effector uncovers an alternative GSK3/β-catenin-regulatory pathway of inflammation
Source: eLife. 2018 Oct 15;7:e39887. doi: 10.7554/eLife.39887 (PMC6214654; doi:10.7554/eLife.39887)
Supplement: Transparent reporting form [file elife-39887-transrepform.docx]

***eLife’s* transparent reporting form**

We encourage authors to provide detailed information *within their submission* to facilitate the interpretation and replication of experiments. Authors can upload supporting documentation to indicate the use of appropriate reporting guidelines for health-related research (see [EQUATOR Network](http://www.equator-network.org/%20)), life science research (see the [BioSharing Information Resource](https://biosharing.org/)), or the [ARRIVE guidelines](http://www.plosbiology.org/article/info:doi/10.1371/journal.pbio.1000412) for reporting work involving animal research. Where applicable, authors should refer to any relevant reporting standards documents in this form.

If you have any questions, please consult our Journal Policies and/or contact us: [editorial@elifesciences.org](mailto:editorial@elifesciences.org).

**Sample-size estimation**

- You should state whether an appropriate sample size was computed when the study was being designed
- You should state the statistical method of sample size computation and any required assumptions
- If no explicit power analysis was used, you should describe how you decided what sample (replicate) size (number) to use

Please outline where this information can be found within the submission (e.g., sections or figure legends), or explain why this information doesn’t apply to your submission:

Sample size for the *Toxoplasma gondii* virulence assay in mice was 3 per

biological replicate. At least 2 biological replicates were performed for each *T. gondii* strain tested. The sample size was chosen based on a

published protocol from the lab (Gay *et al*., 2016) and another group (Nadipuram *et al.,* 2016). All the other experiments were performed at least in triplicate from at least two independent experiments in accordance with the well-established reporting procedures for these assays.

**Replicates**

- You should report how often each experiment was performed
- You should include a definition of biological versus technical replication
- The data obtained should be provided and sufficient information should be provided to indicate the number of independent biological and/or technical replicates
- If you encountered any outliers, you should describe how these were handled
- Criteria for exclusion/inclusion of data should be clearly stated
- High-throughput sequence data should be uploaded before submission, with a private link for reviewers provided (these are available from both GEO and ArrayExpress)

Please outline where this information can be found within the submission (e.g., sections or figure legends), or explain why this information doesn’t apply to your submission:

*Immunofluorescence assays* were performed at least in duplicate (technical replication) and at least in two independent experiments (biological replicates).

The results for the *interaction domain mapping by* *Y2H* were performed in three technical replicates as indicated in the *Materials and Methods* section.

The results for the *Toxoplasma intracellular growth assay* are shown as mean (+/- standard deviation) of three independent replicate experiments. The data value for each independent replicate experiment is taken as the average of three technical replicates. This has been reported in the *Materials and Methods section* as well as in the Figure legends.

The qPCR analyses are shown as mean (+/- standard deviation) of three independent technical replicates. A representative data set of at least two independent biological replicates is shown. This has been reported in the *Materials and Methods section* as well as in the Figure legends.

ELISA assays are shown as mean (+/- standard deviation) of four independent experiments from two biological replicates. This has been reported in the *Materials and Methods section* as well as in the Figure legends.

**Statistical reporting**

- Statistical analysis methods should be described and justified
- Raw data should be presented in figures whenever informative to do so (typically when N per group is less than 10)
- For each experiment, you should identify the statistical tests used, exact values of N, definitions of center, methods of multiple test correction, and dispersion and precision measures (e.g., mean, median, SD, SEM, confidence intervals; and, for the major substantive results, a measure of effect size (e.g., Pearson's r, Cohen's d)
- Report exact p-values wherever possible alongside the summary statistics and 95% confidence intervals. These should be reported for all key questions and not only when the p-value is less than 0.05.

Please outline where this information can be found within the submission (e.g., sections or figure legends), or explain why this information doesn’t apply to your submission:

Detailed information with regards to all statistical analyses performed can be found in the legends for each figure and also in the results text.

Briefly, interpretation of the results of the Y2H screening using the PBS score is described in the *Materials and Methods* section.

For the Gene Set Enrichment Analysis (GSEA), the enrichment scores (FDR q-value) were determined as described in the *Materials and Methods* section and in the legend of Figure 5C.

The significance of the effects of GRA18 on *T. gondii* virulence in mice was determined by Log-rank (Mantel-Cox) test in Figure 4D.

To support our assessment on β-catenin for full induction of *Ccl17*, *Ccl22*, and *Ccl24* by GRA18, *P*-values were calculated using two-tailed unpaired Student’s *t*-test or one-way ANOVA with Bonferroni posttests analysis of variance on the RT-qPCR results in Figures 6E,F.

(For large datasets, or papers with a very large number of statistical tests, you may upload a single table file with tests, Ns, etc., with reference to sections in the manuscript.)

**Group allocation**

- Indicate how samples were allocated into experimental groups (in the case of clinical studies, please specify allocation to treatment method); if randomization was used, please also state if restricted randomization was applied
- Indicate if masking was used during group allocation, data collection and/or data analysis

Please outline where this information can be found within the submission (e.g., sections or figure legends), or explain why this information doesn’t apply to your submission:

No group allocation was applied.

**Additional data files (“source data”)**

- We encourage you to upload relevant additional data files, such as numerical data that are represented as a graph in a figure, or as a summary table
- Where provided, these should be in the most useful format, and they can be uploaded as “Source data” files linked to a main figure or table
- Include model definition files including the full list of parameters used
- Include code used for data analysis (e.g., R, MatLab)
- Avoid stating that data files are “available upon request”
- **Figure 2-figure supplement 1** accompanies the Figure 2. It is an extended version of Figure 2 with graphic representation of the most relevant hits identified by Y2H.
- **Figure 5-figure supplement 1** shows (A) RNA-Seq data about the *T. gondii* transcripts obtained at the GRA18 locus. This panel demonstrates that the whole RNA-Seq assay - from RNA extraction to reads normalization - has worked properly and ascertain that GRA18 is no longer expressed in the *gra18* deleted strain and that GRA18 is overexpressed in the GRA18^+++^ complemented strain when compared to the wild-type parasites. (B) Venn diagram depicting the overlap between the Wnt/β-catenin target genes and those affected in a GRA18-dependent fashion in infected BMDMs. This data shows the lack of overlap between the genes regulated by GRA18 and those regulated by TCF/LEF/β-catenin. (C) the RNA-Seq data published by Saeij’s lab for the GRA18’s specific gene set. This analysis reveals that the ability of GRA18 to induce its target genes in BMDMs in conserved by the different *T. gondii* strains. These supplementary data correspond to a more in-depth analysis of the transcriptomic data presented in the manuscript and therefore accompany the *Results* section describing the figure 5.
- **Figure 5-figure supplement 2** shows the results for GRA18 secretion and export in the host cell in the absence of the aspartyl protease ASP5, an enzyme known to be involved in the export of certain effectors in *Toxoplasma* and *Plasmodium* species. These data accompany the discussion about the results presented in the Figure 1.
- **Supplementary File 1** contains supplementary information about the Strains, Plasmids, Primers, and Oligonucleotides described in the *Materials and Methods* section.
- **Supplementary File 2** contains the entire data set obtained by Yeast-two hybrid screening for the GRA18 interactants described in the *Results* section corresponding to the figure 2.
- **Supplementary File 3** contains the source data for the transcriptomic analysis by Next Generation Sequencing of mouse bone marrow derived macrophages (BMDMs) infected by wild-type and *gra18* mutant strains of *T. gondii* has been deposited on through NCBI GEO with accession number GSE103113 (<http://www.ncbi.nlm.nih.gov/geo/query/acc.cgi?acc=GSE103113>). It contains also additional information about the RNA-Seq experiments: sheets with general information about Illumina NGS and number of reads obtained specifically for *T. gondii* or for murine macrophages (BMDMs) are depicted. Sheets containing specific analysis of the normalized RNA-Seq reads are presented for Pru vs *gra18*, ui vs ui_LPS, and the β-catenin target gene set. These data provide the RPKM values for the genes differentially regulated between different conditions and they accompany the *Materials and Methods* section, the *Result* section for the figure 5, and the *Discussion* section.
- The following source data files have been provided:
- **Figure 3-source data 1:** contains the data source for the Interaction Domain Mapping Assay performed on GRA18 by Y2H presented in figure 3A.

Please indicate the figures or tables for which source data files have been provided:

eLife Sciences Publications, Ltd is a limited liability non-profit non-stock corporation incorporated in the State of Delaware, USA, with company number 5030732, and is registered in the UK with company number FC030576 and branch number BR015634 at the address 1st Floor, 24 Hills Road, Cambridge CB2 1JP | August 2014
